# The Synergistic Effect of Plasminogen Activator Inhibitor-1 (PAI-1) Polymorphisms and Metabolic Syndrome on Coronary Artery Disease in the Korean Population

**DOI:** 10.3390/jpm10040257

**Published:** 2020-11-28

**Authors:** Han Sung Park, Jung-Hoon Sung, Chang Soo Ryu, Jeong Yong Lee, Eun Ju Ko, In Jai Kim, Nam Keun Kim

**Affiliations:** 1 Department of Biomedical Science, College of Life Science, CHA University, Seongnam 13488, Korea; hahnsung@naver.com (H.S.P.); regis2040@nate.com (C.S.R.); smilee3625@naver.com (J.Y.L.); ejko05@naver.com (E.J.K.); 2 Department of Cardiology, CHA Bundang Medical Center, CHA University, Seongnam 13496, Korea; atropin5@cha.ac.kr

**Keywords:** coronary artery disease, *PAI-1* polymorphism, metabolic syndrome

## Abstract

The most common type of cardiovascular disease is coronary artery disease (CAD), in which a plaque builds up inside the coronary arteries that can lead to a complete blockage of blood flow to the heart, resulting in a heart attack. The CAD may be affected by various factors including age, gender, and lipoprotein disposition as well as genetic factors and metabolic syndrome. In this study, we investigated whether three *PAI-1* polymorphisms (−844 G > A, −675 4G > 5G, and +43 G > A) and CAD-related clinical parameters are associated with CAD susceptibility. Genotyping of 463 CAD patients and 401 controls was performed using polymerase chain reaction restriction fragment length polymorphism analysis. We report that the 4G5G genotype (crude odds ratio(COR), 1.392; 95% confidence interval (CI), 1.036–1.871; *p* = 0.028) and dominant model (4G4G vs. 4G5G + 5G5G; COR, 1.401; 95% CI, 1.060–1.850; *p* = 0.018; adjust odds ratio, 1.371; 95% CI, 1.027–1.831; *p* = 0.032) of *PAI-1* −675 polymorphisms were associated with increased CAD risk. Haplotype and genotype combinations of PAI-1 −675 and +43 polymorphisms show an increased risk of CAD according to alterations of the −675 polymorphism allele or genotype. Moreover, the *PAI-1* -675 polymorphisms show a synergistic effect with the metabolic syndrome component of CAD risk. This study suggests that polymorphisms in the *PAI-1* genes along with the metabolic syndrome component of CAD can be useful biomarkers for CAD diagnosis and treatment.

## 1. Introduction

Coronary artery disease (CAD), a cardiovascular disease, is one of the main causes of death in developing countries [1]. CAD is still the leading cause of mortality in Europe, the United States, and Asia [2]. CAD is primarily caused by a buildup of plaque in the coronary artery wall that supplies blood to the heart. Therefore, CAD can weaken the heart muscle and may lead to a serious condition called heart failure that decreases the ability of the heart to pump blood efficiently [3]. This atherosclerotic disease is highly affected by inflammation, higher low-density lipoprotein (LDL) cholesterol, lower high-density lipoprotein (HDL) cholesterol, and plaque formation [4]. However, atherosclerotic disease can also develop at lower LDL-cholesterol levels when risk factors such as age, gender, hypertension, diabetes mellitus, and genetic susceptibility are present [5]. Moreover, the heritability of CAD is estimated at 40 to 50% using an updated genome-wide approach [5], and various studies report that numerous polymorphisms of fibrin clotting and fibrinolysis-related genes are associated with CAD susceptibility [6,7].

Following the formation of a fibrin clot, the fibrinolytic system is initiated by the conversion of plasminogen to plasmin [8]. Plasmin is activated by serine proteases such as tissue plasminogen activator (tPA) and urokinase plasminogen activators (uPA) and contributes to vascular smooth muscle migration and neointimalization through degradation of fibronectin and laminin [9,10]. Moreover, plasmin is involved in the degradation of fibrin and the activation of matrix metalloproteases (MMP), which induce the degradation of elastin and collagen in the extracellular matrix [11]. This fibrinolytic system may be inhibited by regulation of the plasminogen activator inhibitor-1 (*PAI-1*).

The *PAI-1* gene, officially named *SERPINE1*, encodes a member of the serine proteinase inhibitor super family. The *PAI-1* gene is located on chromosome 7 (7q22.1) and PAI-1 is mainly produced by the endothelium. This proteolytic factor is a principal regulatory protein in the fibrinolytic system, and has roles as a main inhibitor of tPA and uPA [12]. An abnormal increase in expression or activity of PAI-1 has been reported to be associated with impaired fibrinolysis [13]. Moreover, several polymorphisms in the *PAI-1* gene may be involved in the alteration of PAI-1 expression and are associated with various diseases.

The +43 G > A polymorphism (rs6092, Ala15Thr) in the first exon of the *PAI-1* gene is associated with plasma insulin levels [14], type 2 diabetes and related metabolic traits [15], and osteonecrosis [16]. The −844 A > G polymorphism (rs2227631) is located in the promotor region of the *PAI-1* gene, leads to increased PAI-1 protein levels, and is associated with osteonecrosis of the femoral head, osteoporotic vertebral compression fracture [17], and acute coronary syndrome [18,19]. The −675 4G > 5G polymorphism (rs1799762), located in the *PAI-1* promotor region, is also reported to be associated with various atherosclerotic diseases including venous thromboembolism [20], ischemic stroke [21], carotid artery stenosis [22], renal artery stenosis [23], and coronary artery disease [24]. Moreover, these three polymorphisms are reported to be associated with plasma PAI-1 levels [25,26]. Therefore, we designed a genetic epidemiological study of the three most extensively studied polymorphisms of *PAI-1* to investigate the association between *PAI-1* and CAD in Korean populations.

## 2. Results

### 2.1. Clinical Characteristics of the Study Participants

Baseline characteristics of the CAD patients and controls are presented in Table 1. The age and gender of CAD patients and controls were statistically matched. The mean age of CAD patients (mean ± standard deviation (SD), 60.40 ± 11.68) and control participants (mean ± SD, 60.02 ± 11.46) were not significantly different. Moreover, the male ratio of CAD patients and controls was not significantly different (202/463 (43.6%) and 171/401 (42.6%), respectively). The mean body mass index (BMI) of CAD patients (mean ± SD, 25.09 ±3.59) was significantly higher than controls (mean ± SD, 24.19 ± 3.31). Hypertension was significantly higher in CAD patients than controls (248 (53.6%) and 149/401 (37.2%), *p* < 0.0001). Additionally, the ratios of diabetes mellitus (*p* < 0.0001) and metabolic syndrome (MetS) (*p* < 0.0001) in CAD patients and controls were significantly different. The clinical parameters of total cholesterol (*p* = 0.004) and creatinine (*p* = 0.0004) were significantly different between CAD patients and controls. However, triglyceride (*p* = 0.061), HDL-cholesterol (*p* = 0.086), LDL-cholesterol (*p* = 0.184), homocysteine (*p* = 0.142), vitamin B12 (*p* = 0.833), and folate (*p* = 0.264) were not significantly different between the two groups.

### 2.2. Genotype Frequencies Comparison Analysis

To evaluate the association of the three polymorphisms (*PAI-1* −844 G > A, *PAI-1* −675 4G > 5G, and *PAI-1* +43 G > A) with CAD susceptibility, the genotype frequencies between CAD patients and control participants were compared and summarized in Table 2. The frequency of the *PAI-1* −675 4G > 5G polymorphism was significantly different in the 4G5G genotype and dominant model (4G4G vs. 4G5G + 5G5G). The crude odds ratio (COR) and *p*-value of the dominant model were 1.401 and 0.018, respectively. Moreover, the significance of the dominant model was maintained in adjusted statistical analysis (adjusted odds ratio (AOR), 1.371; *p* = 0.032) using age, gender, hypertension, diabetes mellitus, hyperlipidemia, and smoking status.

Genotype analysis of the MetS subgroup was performed to investigate whether the associations of the three polymorphisms change according to the existence or nonexistence of MetS (results summarized in Table 3). The CAD patients and controls were divided into four subgroups according to MetS, and the genotype frequencies of controls with non-MetS were compared. The CAD patients in the MetS groups in the dominant model of *PAI-1* −675 4G > 5G polymorphism (AOR, 1.519; *p* = 0.045) are associated with increased CAD susceptibility.

### 2.3. Haplotype and Genotype Combination Analysis

Haplotype and genotype combination analysis was performed to confirm the combined effect of the three SNPs. The results of haplotype and genotype combination analysis were summarized in Table 4 and Table 5, respectively. In haplotype analysis of the *PAI-1* −844 G > A/*PAI-1* −675 4G > 5G/*PAI-1* +43 G > A polymorphisms, G-4G-A (OR, 0.118; *p* < 0.0001), A-4G-A (OR, 0.019; *p* < 0.0001), G-5G-A (OR, 1.989; *p* = 0.005), and A-5G-A (OR, 4.728; *p* = 0.002) are associated with CAD susceptibility. In haplotypes of two SNPs, A-5G (OR, 6.503; *p* < 0.0001) of *PAI-1* −844 G > A/*PAI-1* −675 4G > 5G, 4G-A (OR, 0.059; *p* < 0.0001) and 5G-A (OR, 2.276; *p* = 0.0002) of *PAI-1* −675/*PAI-1* +43, and A-A (OR, 0.118; *p* = 0.0001) of *PAI-1* −844/*PAI-1* +43 are associated with CAD risk.

In genotype combination analysis, the 4G4G/GA (AOR, 0.062; *p* = 0.0001) of *PAI-1* −675 4G > 5G/*PAI-1* +43 G > A shows a decreased risk of CAD. In contrast, AA/4G5G (AOR, 13.157; *p* = 0.022) of *PAI-1* −844 G > A/*PAI-1* −675 4G > 5G, GG/GA (AOR, 2.215; *p* = 0.010) of *PAI-1* −844 G > A/*PAI-1* +43 G > A, 4G5G/GA (AOR, 2.089; *p* = 0.017) and 5G5G/GA (AOR, 2.558; *p* = 0.010) of *PAI-1* −675 4G > 5G/*PAI-1* +43 G > A genotype combinations are associated with a highly increased CAD risk.

Interestingly, when the 4G to 5G alteration of the *PAI-1* −675 polymorphism is combined with the GA genotype or A allele of *PAI-1* +34 polymorphism, there is an increased OR. When the GA genotype of the *PAI-1* +43 G > A polymorphism is in combination with the *PAI-1* −675 4G > 5G/*PAI-1* +43 G > A, the alterations of *PAI-1* −675 genotype leads to an increase in CAD risk. This pattern is maintained in the *PAI-1* −675/*PAI-1* +43 haplotype. 

### 2.4. Synergistic Effect of PAI-1 Polymorphisms with Clinical Parameter

**Figure 1:**
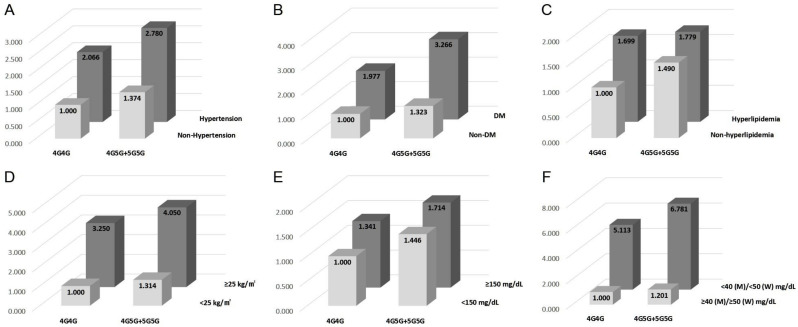
Synergistic effect of *PAI-1* −675 4G > 5G polymorphism with metabolic syndrome-related clinical parameters. (**A**–**F**) panels show AOR of *PAI-1* −675 4G > 5G with metabolic syndrome-related clinical parameters including hypertension (**A**), diabetes mellitus (**B**), hyperlipidemia (**C**), body mass index (**D**), triglyceride levels (**E**), and high-density lipoprotein cholesterol (**F**), respectively.

We investigated the synergistic effect of the *PAI-1* polymorphisms with clinical parameters. Various clinical parameters showed synergistic effects with *PAI-1* polymorphisms (Supplementary Table S1). The MetS-related clinical parameters combined with the *PAI-1* −675 polymorphism are highly associated with an increased risk of CAD (Figure 1). The AORs of *PAI-1* −675 4G5G + 5G5G group when combined with each parameters including hypertension, DM, hyperlipidemia, ≥25 kg/m^2^ of BMI, ≥150 mg/dL of triglyceride levels, and <40 (male)/<50 (female) mg/dL of HDL-cholesterol levels are 2.780 (*p* < 0.0001), 3.266 (*p* < 0.0001), 1.779 (*p* = 0.011), 4.050 (*p* < 0.0001), 1.714 (*p* = 0.011), and 6.781 (*p* < 0.0001).

## 3. Discussion

In this study, we investigated the association of three polymorphisms in the *PAI-1* gene with differences in susceptibility of CAD. The polymorphism *PAI-1* +43 G > A is a missense variant in the first exon of the *PAI-1* gene. The other two polymorphisms, *PAI-1* −675 4G > 5G and *PAI-1* −844 G > A, are in the promoter region of the *PAI-1* gene. Therefore, these three polymorphisms may act as functional polymorphisms that affect the regulation of gene expression and fibrinolysis.

In age and gender matched groups of CAD patients and controls, the genotype frequency comparison analysis shows that the 4G5G + 5G5G genotypes are associated with the CAD risk when compared with the 4G4G genotype in a dominant model of the *PAI-1* -675 4G > 5G polymorphism. The association of the −675 polymorphism is more powerful when combined with the other *PAI-1* polymorphisms. In the *PAI-1* −675 4G > 5G/*PAI-1* +43 G > A genotype combination analysis, the *PAI-1* −675 4G5G/*PAI-1* +43 G > A combination shows that the susceptibility of CAD was gradually increased when the genotype of *PAI-1* +43 G > A was GA and the genotypes of *PAI-1* −675 4G > 5G changed from 4G4G to 5G5G. These patterns of association are also shown in the two and three allele combination analyses. The CAD susceptibility is greatly increased when the allele of *PAI-1* +43 G > A is A and the allele of *PAI-1* −675 4G > 5G is changed from 4G to 5G in the two allele combination analysis. In the three allele combination analysis, the CAD risk increases according to the alteration of −675 4G > 5G, when the −844 G > A is the G allele and +43 G > A is the A allele.

The serine proteinase inhibitor super family E member 1 is encoded by the *PAI-1* gene. The PAI-1 is mainly produced by endothelial cells and stored at platelet. Secreted PAI-1 from alpha-granules of activated platelets and endothelial cells is incorporated into the coagulation process and plays a key role in thrombolysis resistance [27,28]. Moreover, the PAI-1 regulates the initiation of fibrinolytic processes through the inhibition of tPA and uPA. The *PAI-1* polymorphisms were studied in various groups, populations, and diseases. The *PAI-1* −675 4G > 5G polymorphism is reported to be associated with increased susceptibility of atherosclerotic diseases in various populations. Numerous previous studies report that the *PAI-1* −675 4G > 5G polymorphism overlaps with the enhancer box (E-box) which is recognized and bound by transcription factors to initiate gene transcription [29]. Moreover, the *PAI-1* −675 4G > 5G polymorphism affects *PAI-1* expression levels and various pathways including the thrombolytic and fibrinolytic pathway, and is associated with CAD [30]. Various meta-analysis of the *PAI-1* −675 4G > 5G polymorphism show that the 5G allele is associated with increased CAD susceptibility [31,32,33]. 

Various studies have reported that the components of MetS and atherosclerotic diseases containing CAD are closely linked [34,35,36,37]. According to ATP III criteria, diagnosis of MetS is based on the presence of three or more of the five criteria including waist circumference (WC) > 102 cm in men and > 88 cm in women, high blood pressure (BP ≥ 130/85), high triglyceride (TG ≥ 150), high fasting blood sugar (FBS ≥ 110), and low HDL-cholesterol (< 40 in men and < 50 in women) [38]. Table 3 shows that the 4G5G + 5G5G genotypes of the *PAI-1* −675 variant in the MetS CAD group show increased AOR when compared to 4G4G in non-MetS controls. Interestingly, the *PAI-1* −675 variant and risk factors of MetS have a synergistic effect for increased susceptibility of CAD (Figure 1). Each group that has the *PAI-1* −675 4G5G + 5G5G genotype and the six risk conditions for MetS including hypertension, DM, hyperlipidemia, BMI ≥ 25 kg/m2, TG ≥ 150 mg/dL, and HDL < 40 mg/dL (male) and <50 mg/dL (female) exhibit a significantly increased OR (AOR = 2.780, 3.266, 1.779, 4.050, 1.714 and 6.781, respectively) when compared to the *PAI-1*−675 4G4G group with non-MetS conditions. 

This study evaluates whether the three polymorphisms (*PAI-1* −675 4G > 5G, *PAI-1* −844 G > A, and *PAI-1* +43 G > A) that may affect PAI-1 expression or activity are associated with susceptibility of CAD in the Korean population. The *PAI-1* −675 4G > 5G polymorphism is associated with CAD and the MetS-CAD subgroup. In combination analysis, some alleles and genotype combinations including the *PAI-1* −675 4G > 5G polymorphism are associated with highly increased CAD susceptibility. Moreover, the *PAI-1* −675 polymorphism and some conditions that may increase the risk of MetS show synergistic effects on CAD risk. This finding could be applied to identify new CAD prognostic biomarkers using the *PAI-1* −675 polymorphism when combined with other *PAI-1* polymorphisms and the component of MetS.

## 4. Materials and Methods

### 4.1. Study Participants

Blood samples were collected from 463 patients with CAD (age; mean ± standard deviation (SD): 60.40 ± 11.68 years) and 401 healthy control participants (age; mean ± SD: 60.02 ± 11.46 years). The participants were recruited from the Department of Cardiology of CHA Bundang Medical Center (Seongnam, South Korea) between 2014 and 2016. All participants gave written informed consent to this study, which was approved by the Institutional Review Board of CHA Bundang Medical Center (IRB number: 2013-10-114), and all study protocols followed the recommendations of the Declaration of Helsinki. In total, 463 patients with CAD were referred from the Department of Cardiology of CHA Bundang Medical Center, CHA University.

All patients had stenosis of more than 50% in at least one of the main coronary arteries or their major branches, which was confirmed by coronary angiography. To avoid issues in blood testing caused by various medical treatments, exclusion criteria included history of cardiac arrest and life expectancy <1 year. Diagnoses were made by coronary angiography and were confirmed by at least one independent experienced cardiologist.

We randomly selected 401 gender and age matched control participants from patients presented at the Department of Cardiology at the CHA Bundang Medical Center during the same period for comprehensive health check-up, including biochemical testing and cardiological examination. The control participants that had a history of angina symptoms or myocardial infarction and showed T wave inversion on electrocardiography were excluded in control subjects.

In this study, the criterion of hypertension was defined as systolic pressure ≥130mmHg and diastolic pressure ≥85 mmHg and included patients currently taking hypertensive medications. Diabetes mellitus was defined as a fasting plasma glucose level ≥110 mg/dL and included patients taking diabetic medications. Hyperlipidemia was defined as a high fasting serum total cholesterol (TC) level (≥150 mg/dL) or an anti-hyperlipidemic agent treatment history. Smoking status refers to patients who currently smoke. 

### 4.2. Blood Biochemical Analyses

Blood was collected in anticoagulant tubes after 12 h of fasting. To separate plasma from whole blood, samples were centrifuged for 15 min at 1000× *g*. The plasma levels of homocysteine, folate, TC, TG, HDL-cholesterol, and LDL-cholesterol were determined [3].

### 4.3. Genetic Analyses

DNA was extracted from leukocytes in peripheral blood using G-dex II Genomic DNA Extraction kit (iNtRON Biotechnology, Inc., Seongnam, Korea), according to the manufacturer’s instructions. Polymerase chain reaction (PCR) restriction fragment length polymorphism (RFLP) assays was performed to analyze the *PAI-1* −884 G > A, *PAI-1* −675 4G > 5G and *PAI-1* +43 G > A polymorphisms [39]. To amplify the three polymorphic regions, three primer sets were used (Supplementary Table S2). The PCR conditions were based on the following steps: pre-denaturation was performed at 95 °C for 10 min, followed by 35 cycles with denaturation at 95 °C for 30 s, annealing at each optimized temperature for 30 s, extension at 72 °C for 45 s, and final extension was carried out at 72 °C for 7 min. The PCR product was loaded in 3% agarose gel stained by nucleic acid staining solution and visualized using ultraviolet illuminator after 16 h of enzyme restriction.

### 4.4. Statistical Analysis

In the clinical characteristics analysis of CAD patients and control participants, the student’s *t*-test for continuous variables and the Chi-square test for categorical variables were used. To estimate the relative risk of the *PAI-1* genotype for CAD occurrence, logistic regression analyses were performed using age, gender, hypertension, Diabetes mellitus, hyperlipidemia, and smoking status. For allele combination analysis, the Chi square test and Fisher’s exact test were used. *p* < 0.05 was considered to indicate a statistically significant difference and false discovery rate (FDR) *p*-values were calculated. Analyses were performed using GraphPad Prism 4.0 (GraphPad Software, Inc., San Diego, CA, USA), StatsDirect Statistical Software Version 2.4.4 (StatsDirect Ltd., Altrincham, UK), and MedCalc (Version 7.4 for Windows; MedCalc, Ostend, Belgium). HAPSTAT software was used to estimate the frequencies of allele combinations of the *PAI-1* polymorphisms. Current versions of the HAPSTAT software (v.3.0) are available from www.bios.unc.edu/~lin/hapstat/. 

## Figures and Tables

**Table 1:** Baseline characteristics between CAD and controls.

Characteristic	Controls (*n* = 401)	CAD Patients (*n* = 463)	*p*
Age (years, mean ± SD)	60.02 ± 11.46	60.40 ± 11.68	0.703
Male (%)	171 (42.6)	202 (43.6)	0.771
BMI (kg/m^2^, mean ± SD)	24.19 ± 3.31	25.09 ± 3.59	0.001
Hypertension (n, %)	149 (37.2)	248 (53.6)	<0.0001
Diabetes mellitus (n, %)	48 (12.0)	118 (25.5)	<0.0001
Hyperlipidemia (n, %)	88 (21.9)	119 (25.7)	0.174
Smoking (n, %)	141 (35.2)	148 (32.0)	0.403
Metabolic syndrome (n, %)	62 (15.5)	209 (45.1)	<0.0001
Total cholesterol (mg/dL, mean ± SD)	191.76 ± 37.43	185.65 ± 45.97	0.004
Triglyceride (mg/dL, mean ± SD)	143.25 ± 88.91	155.03 ± 92.46	0.061
HDL-cholesterol (mg/dL, mean ± SD)	46.01 ± 14.00	43.71 ± 11.35	0.086
LDL-cholesterol (mg/dL, mean ± SD)	116.16 ± 40.84	111.42 ± 39.17	0.184
Homocysteine (μmol/L, mean ± SD)	9.79 ± 4.18	9.65 ± 4.85	0.142
Vitamin B12 (pg/mL, mean ± SD)	675.99 ± 259.21	710.04 ± 346.17	0.833
Folate (nmol/L, mean ± SD)	8.88 ± 7.99	8.27 ± 7.58	0.264
Creatinine (mg/dL, mean ± SD)	0.94 ± 0.23	1.48 ± 6.65	0.0004

Note: CAD, coronary artery disease; BMI, body mass index; HDL, high-density lipoprotein; LDL, low-density lipoprotein.

**Table 2:** Genotype frequencies of *PAI-1* polymorphisms in CAD and controls.

Genotypes	Controls (*n* = 401)	CAD (*n* = 463)	COR (95% CI)	*p*	FDR-*p*	AOR (95% CI)	*p*	FDR-*p*
*PAI-1* −844 G > A								
GG	135 (33.7)	167 (36.1)						
GA	196 (48.9)	214 (46.2)	0.883 (0.655–1.189)	0.412	0.525	0.820 (0.601–1.119)	0.211	0.352
AA	70 (17.5)	82 (17.7)	0.947 (0.640–1.401)	0.785	0.785	0.960 (0.633–1.455)	0.847	0.847
Dominant (GG vs. GA + AA)			0.900 (0.679–1.191)	0.460	0.690	0.853 (0.637–1.143)	0.287	0.478
Recessive (GG + GA vs. AA)			1.018 (0.716–1.446)	0.922	0.922	1.085 (0.755–1.558)	0.661	0.826
HWE-*P*	0.937	0.351						
*PAI-1*-675 4G > 5G								
4G4G	162 (40.4)	151 (32.6)						
4G5G	178 (44.4)	231 (49.9)	1.392 (1.036–1.871)	0.028	0.084	1.342 (0.987–1.824)	0.060	0.151
5G5G	61 (15.2)	81 (17.5)	1.425 (0.956–2.124)	0.083	0.124	1.503 (0.992–2.276)	0.054	0.272
Dominant (4G4G vs. 4G5G + 5G5G)			1.401 (1.060–1.850)	0.018	0.054	1.371 (1.027–1.831)	0.032	0.127
Recessive (4G4G + 4G5G vs. 5G5G)			1.182 (0.822–1.699)	0.367	0.5505	1.259 (0.866–1.830)	0.227	0.568
HWE-*P*	0.297	0.649						
*PAI-1* + 43 G > A								
GG	333 (83.0)	382 (82.5)						
GA	62 (15.5)	80 (17.3)	1.125 (0.783–1.617)	0.525	0.525	1.229 (0.843–1.791)	0.283	0.354
GG	6 (1.5)	1 (0.2)	0.145 (0.017–1.213)	0.075	0.125	0.191 (0.022–1.633)	0.131	0.326
Dominant (GG vs. GA + GG)			1.038 (0.729–1.480)	0.835	0.835	1.145 (0.792–1.654)	0.472	0.589
Recessive (GG + GA vs. GG)			0.143 (0.017–1.189)	0.072	0.216	0.185 (0.022–1.589)	0.124	0.568
HWE-*P*	0.123	0.13						

CAD, coronary artery disease; COR, crude odds ratio; CI, confidence interval; FDR, false discovery rate; AOR, adjusted odds ratio. AOR: Adjusted by age, gender, hypertension, diabetes mellitus, hyperlipidemia, and smoking status.

**Table 3:** Genotype frequencies of *PAI-1* polymorphisms according to metabolic syndrome (MetS).

Genotypes	Non-MetS Controls (*n* = 296)	MetS Control (*n* = 105)	AOR (95% CI)	*p*	Non-MetS CAD (*n* = 189)	AOR (95% CI)	*p*	MetS CAD (*n* = 274)	AOR (95% CI)	*p*
*PAI-1* −844 G > A										
GG	103 (34.8)	32 (30.5)	1.000 (reference)		68 (36.0)	1.000 (reference)		99 (36.1)	1.000 (reference)	
GA	137 (46.3)	59 (56.2)	1.003 (0.553–1.820)	0.991	86 (45.5)	0.907 (0.590–1.395)	0.658	128 (46.7)	0.872 (0.562–1.351)	0.539
AA	56 (18.9)	14 (13.3)	0.786 (0.366–1.687)	0.537	35 (18.5)	0.923 (0.533–1.596)	0.773	47 (17.2)	0.800 (0.448–1.429)	0.451
Dominant (GG vs. GA + AA)			0.939 (0.541–1.630)	0.824		0.914 (0.613–1.362)	0.658		0.854 (0.569–1.281)	0.445
Recessive (GG + GA vs. AA)			0.750 (0.372–1.515)	0.423		1.024 (0.631–1.661)	0.925		0.848 (0.510–1.413)	0.528
*PAI-1* −675 4G > 5G										
4G4G	124 (41.9)	38 (36.2)	1.000 (reference)		68 (36.0)	1.000 (reference)		83 (30.3)	1.000 (reference)	
4G5G	130 (43.9)	48 (45.7)	1.103 (0.626–1.945)	0.735	87 (46.0)	1.121 (0.735–1.712)	0.596	144 (52.6)	1.487 (0.967–2.288)	0.071
5G5G	42 (14.2)	19 (18.1)	1.739 (0.815–3.710)	0.152	34 (18.0)	1.588 (0.906–2.785)	0.107	47 (17.2)	1.694 (0.937–3.063)	0.081
Dominant (4G4G vs. 4G5G + 5G5G)			1.233 (0.730–2.083)	0.434		1.233 (0.832–1.827)	0.296		1.519 (1.010–2.285)	0.045
Recessive (4G4G + 4G5G vs. 5G5G)			1.617 (0.822–3.181)	0.164		1.495 (0.899–2.484)	0.121		1.347 (0.794–2.283)	0.269
*PAI-1* +43 G > A										
GG	241 (81.4)	92 (87.6)	1.000 (reference)		157 (83.1)	1.000 (reference)		225 (82.1)	1.000 (reference)	
GA	51 (17.2)	11 (10.5)	0.552 (0.252–1.211)	0.138	31 (16.4)	0.930 (0.560–1.545)	0.780	49 (17.9)	1.181 (0.711–1.962)	0.521
GG	4 (1.4)	2 (1.9)	2.008 (0.262–5.406)	0.503	1 (0.5)	0.502 (0.055–4.590)	0.542	0 (0.0)	N/A	0.998
Dominant (GG vs. GA + GG)			0.629 (0.301–1.314)	0.217		0.906 (0.551–1.489)	0.696		1.106 (0.670–1.824)	0.695
Recessive (GG + GA vs. GG)			2.083 (0.280–5.476)	0.473		0.541 (0.059–4.931)	0.586		N/A	0.998

Note: MetS, metabolic syndrome; AOR, adjusted odds ratio; CI, confidence interval; CAD, coronary artery disease. AOR: Adjusted by age, gender, hypertension, diabetes mellitus, hyperlipidemia, and smoking status.

**Table 4:** Haplotype of the *PAI-1* polymorphisms in coronary artery disease.

Haplotype	Controls (*2n* = 802)	CAD (*2n* = 926)	OR (95% CI)	*p*	*FDR-p*
*PAI-1* −844 G > A/*PAI-1* −675 4G > 5G/*PAI-1* +43 G > A					
G-4G-G	148 (18.5)	180 (19.4)	1.000 (reference)		
G-4G-A	21 (2.7)	3 (0.3)	0.118 (0.034–0.402)	<0.0001	0.0004
G-5G-G	265 (33.0)	291 (31.4)	0.903 (0.687–1.187)	0.486	0.567
G-5G-A	31 (3.9)	75 (8.1)	1.989 (1.241–3.188)	0.005	0.009
A-4G-G	311 (38.8)	350 (37.9)	0.925 (0.709–1.207)	0.588	0.588
A-4G-A	21 (2.7)	0 (0.0)	0.019 (0.001–0.319)	<0.0001	0.0004
A-5G-G	4 (0.5)	23 (2.5)	4.728 (1.599–13.980)	0.002	0.005
A-5G-A	0 (0.0)	4 (0.5)	7.404 (0.395–138.700)	0.132	0.185
*PAI-1* −844 G > A/*PAI-1* −675 G > A4G > 5G					
G-4G	170 (21.2)	183 (19.7)	1.000 (reference)		
G-5G	296 (36.9)	365 (39.5)	1.146 (0.884–1.484)	0.321	0.482
A-4G	332 (41.4)	350 (37.8)	0.979 (0.757–1.267)	0.896	0.896
A-5G	4 (0.5)	28 (3.0)	6.503 (2.234–18.930)	<0.0001	0.0003
*PAI-1* −844 G > A/*PAI-1* +43 G > A					
G-G	413 (51.6)	472 (50.9)	1.000 (reference)		
G-A	53 (6.6)	76 (8.2)	1.255 (0.863–1.825)	0.257	0.386
A-G	315 (39.2)	372 (40.2)	1.033 (0.846–1.262)	0.760	0.760
A-A	21 (2.7)	6 (0.6)	0.250 (0.100–0.626)	0.002	0.006
*PAI-1* −675 4G > 5G/*PAI-1* +43 G > A					
4G-G	458 (57.1)	530 (57.2)	1.000 (reference)		
4G-A	44 (5.5)	3 (0.3)	0.059 (0.018–0.191)	<0.0001	0.0003
5G-G	270 (33.6)	314 (33.9)	1.005 (0.819–1.234)	0.962	0.962
5G-A	30 (3.8)	79 (8.5)	2.276 (1.468–3.529)	0.0002	0.0003

Note: CAD, coronary artery disease; OR, odds ratio; CI, confidence interval; FDR, false discovery rate.

**Table 5:** Genotype combinations of the *PAI-1* polymorphisms in coronary artery disease.

Combination	Controls (*n* = 401)	CAD Patients (*n* = 463)	AOR (95% CI)	*p*
*PAI-1* −844 G > A/*PAI-1* −675 4G > 5G				
GG/4G4G	24 (6.0)	23 (5.0)	1.000 (reference)	
GG/4G5G	52 (13.0)	71 (15.3)	1.566 (0.740–3.312)	0.241
GG/5G5G	59 (14.7)	73 (15.8)	1.429 (0.687–2.974)	0.339
GA/4G4G	69 (17.2)	60 (13.0)	0.886 (0.434–1.808)	0.739
GA/4G5G	125 (31.2)	147 (31.7)	1.223 (0.633–2.362)	0.549
GA/5G5G	2 (0.5)	7 (1.5)	3.052 (0.486–19.172)	0.234
AA/4G4G	69 (17.2)	68 (14.7)	1.102 (0.533–2.279)	0.793
AA/4G5G	1 (0.2)	13 (2.8)	13.157 (1.463–118.330)	0.022
AA/5G5G	0 (0.0)	1 (0.2)	N/A	N/A
*PAI-1* −844 G > A/*PAI-1* +43 G > A				
GG/GG	106 (26.4)	120 (25.9)	1.000 (reference)	
GG/GA	25 (6.2)	46 (9.9)	2.215 (1.213–4.042)	0.010
GG/AA	4 (1.0)	1 (0.2)	0.269 (0.028–2.556)	0.253
GA/GG	167 (41.6)	183 (39.5)	0.942 (0.665–1.335)	0.737
GA/GA	27 (6.7)	31 (6.7)	1.001 (0.542–1.850)	0.997
GA/AA	2 (0.5)	0 (0.0)	N/A	N/A
AA/GG	60 (15.0)	79 (17.1)	1.291 (0.819–2.036)	0.271
AA/GA	10 (2.5)	3 (0.6)	0.273 (0.069–1.088)	0.066
AA/AA	0 (0.0)	0 (0.0)	N/A	N/A
*PAI-1* −675 4G > 5G/*PAI-1* +43 G > A				
4G4G/GG	131 (32.7)	149 (32.2)	1.000 (reference)	
4G4G/GA	30 (7.5)	2 (0.4)	0.062 (0.014–0.269)	0.0001
4G4G/AA	1 (0.2)	0 (0.0)	N/A	N/A
4G5G/GG	156 (38.9)	185 (40.0)	1.003 (0.722–1.394)	0.985
4G5G/GA	19 (4.7)	46 (9.9)	2.089 (1.142–3.824)	0.017
4G5G/AA	3 (0.7)	0 (0.0)	N/A	N/A
5G5G/GG	46 (11.5)	48 (10.4)	0.867 (0.531–1.416)	0.568
5G5G/GA	13 (3.2)	32 (6.9)	2.558 (1.252–5.224)	0.010
5G5G/AA	2 (0.5)	1 (0.2)	0.630 (0.054–7.330)	0.712

Note: CAD, coronary artery disease; AOR, adjusted odds ratio; CI, confidence interval. AOR: adjusted by age, gender, hypertension, diabetes mellitus, hyperlipidemia, and smoking status.

## Supplementary Materials

Supplementary materials can be found at https://www.mdpi.com/2075-4426/10/4/257/s1, Table S1: Synergic effect of PAI-1 polymorphisms with clinical risk factor, Table S2: Information of PAI-1 polymorphism for PCR-RFLP analysis.
