# Supplementary material for: The Synergistic Effect of Plasminogen Activator Inhibitor-1 (PAI-1) Polymorphisms and Metabolic Syndrome on Coronary Artery Disease in the Korean Population
Source: J Pers Med. 2020 Nov 28;10(4):257. doi: 10.3390/jpm10040257 (PMC7711432; doi:10.3390/jpm10040257)
Supplement: Supplementary file 1 [file jpm-10-00257-s001.pdf]

Supplementary Table 1. Synergic effect of *PAI-1* polymorphisms with clinical risk factor.

Characteristics	*PAI-1* -844 GG	*PAI-1* -844 GA+AA	*PAI-1* -675 4G4G	*PAI-1* -675 4G5G+5G5G	*PAI-1* +43 GG	*PAI-1* +43 GA+AA
**Gender**						
Male	1.000 (reference)	0.876 (0.557 - 1.379)	1.000 (reference)	1.307 (0.834 - 2.050)	1.000 (reference)	1.054 (0.593 - 1.871)
Female	0.722 (0.432 - 1.208)	0.617 (0.387 - 0.985)	0.831 (0.513 - 1.345)	1.132 (0.730 - 1.756)	0.872 (0.633 - 1.201)	0.976 (0.591 - 1.613)
**Age**						
<61	1.000 (reference)	0.954 (0.626 - 1.453)	1.000 (reference)	1.337 (0.876 - 2.041)	1.000 (reference)	1.206 (0.723 - 2.012)
≥61	1.054 (0.632 - 1.757)	0.749 (0.490 - 1.146)	0.916 (0.571 - 1.469)	1.237 (0.808 - 1.892)	0.906 (0.659 - 1.245)	1.148 (0.659 - 2.000)
**Hypertension**						
No	1.000 (reference)	0.946 (0.639 - 1.402)	1.000 (reference)	1.374 (0.925 - 2.042)	1.000 (reference)	1.469 (0.913 - 2.361)
Yes	**2.157 (1.291 - 3.602)**	**1.755 (1.163 - 2.648)**	**2.066 (1.269 - 3.363)**	**2.780 (1.823 - 4.237)**	2.110 (1.535 - 2.899)	1.773 (0.994 - 3.163)
**Diabetes mellitus**						
No	1.000 (reference)	0.948 (0.686 - 1.310)	1.000 (reference)	1.323 (0.963 - 1.818)	1.000 (reference)	1.119 (0.752 - 1.667)
Yes	**4.053 (1.964 - 8.363)**	**1.717 (1.044 - 2.825)**	**1.977 (1.011 - 3.867)**	**3.266 (2.004 - 5.324)**	**2.274 (1.517 - 3.407)**	**3.200 (1.132 - 9.041)**
**Hyperlipidemia**						
No	1.000 (reference)	0.920 (0.659 - 1.286)	1.000 (reference)	**1.490 (1.066 - 2.083)**	1.000 (reference)	0.870 (0.566 - 1.337)
Yes	1.800 (0.983 - 3.297)	1.152 (0.744 - 1.782)	1.699 (0.980 - 2.943)	**1.779 (1.138 - 2.780)**	1.079 (0.751 - 1.550)	**3.121 (1.458 - 6.681)**
**Smoking**						
No	1.000 (reference)	0.867 (0.600 - 1.253)	1.000 (reference)	1.409 (0.981 - 2.024)	1.000 (reference)	1.098 (0.693 - 1.741)
Yes	0.964 (0.522 - 1.780)	0.736 (0.442 - 1.227)	0.939 (0.542 - 1.627)	0.974 (0.595 - 1.594)	0.840 (0.577 - 1.220)	1.414 (0.745 - 2.684)
**BMI**						
<25 kg/m^2	**1.000 (reference)**	0.930 (0.640 - 1.351)	1.000 (reference)	1.314 (0.910 - 1.896)	1.000 (reference)	0.931 (0.585 - 1.481)
≥25 kg/m^2	**3.426 (1.999 - 5.870)**	**2.833 (1.811 - 4.431)**	**3.250 (1.909 - 5.532)**	**4.050 (2.626 - 6.245)**	**2.891 (2.067 - 4.045)**	**5.380 (2.671 - 10.837)**
**Total Cholesterol**						
<200 mg/dl	1.000 (reference)	0.954 (0.665 - 1.369)	1.000 (reference)	1.464 (1.015 - 2.112)	1.000 (reference)	0.871 (0.552 - 1.372)
≥200 mg/dl	0.903 (0.486 - 1.681)	0.709 (0.433 - 1.162)	0.753 (0.426 - 1.331)	0.947 (0.566 - 1.583)	0.726 (0.497 - 1.062)	1.465 (0.706 - 3.039)
**Triglycerides**						
<150 mg/dL	1.000 (reference)	0.802 (0.551 - 1.167)	1.000 (reference)	1.446 (1.001 - 2.089)	1.000 (reference)	1.081 (0.673 - 1.737)
≥150 mg/dL	1.005 (0.600 - 1.684)	1.190 (0.773 - 1.832)	1.341 (0.811 - 2.218)	1.714 (1.133 - 2.592)	1.205 (0.870 - 1.667)	1.350 (0.739 - 2.468)
**HDL**						
≥40(M)/≥50(W) mg/dL	1.000 (reference)	0.943 (0.632 - 1.407)	1.000 (reference)	1.201 (0.815 - 1.771)	1.000 (reference)	1.213 (0.740 - 1.986)
<40(M)/<50(W) mg/dL	**5.999 (3.423 - 10.514)**	**4.646 (2.961 - 7.289)**	**5.1137 (2.993 - 8.738)**	**6.781 (4.365 - 10.533)**	**5.561 (3.925 - 7.879)**	**5.675 (2.963 - 10.869)**
**LDL**						
< 130 mg/dL	1.000 (reference)	0.880 (0.557 - 1.390)	1.000 (reference)	1.269 (0.800 - 2.012)	1.000 (reference)	1.216 (0.644 - 2.298)
≥130 mg/dL	0.824 (0.301 - 2.257)	0.565 (0.271 - 1.179)	1.169 (0.458 - 2.985)	0.734 (0.353 - 1.525)	1.068 (0.586 - 1.948)	2.722 (0.999 - 7.416)
**Vitamin B_{12} †**						
>434 pg/mL	1.000 (reference)	0.884 (0.651 - 1.199)	1.000 (reference)	1.282 (0.949 - 1.731)	1.000 (reference)	1.166 (0.796 - 1.708)
≤434 pg/mL	**0.278 (0.093 - 0.829)**	**0.171 (0.078 - 0.377)**	N/A	0.467 (0.234 - 0.933)	**0.254 (0.134 - 0.482)**	**0.096 (0.011 - 0.809)**
**Folate†**						

>3.79 nmol/L	1.000 (reference)	0.827 (0.603 - 1.133)	1.000 (reference)	1.337 (0.978 - 1.828)	1.000 (reference)	1.259 (0.852 - 1.861)
≤3.79 nmol/L	2.069 (0.885 - 4.836)	**1.837 (1.042 - 3.240)**	1.870 (0.906 - 3.859)	**2.974 (1.659 - 5.333)**	2.289 (1.425 - 3.678)	1.909 (0.597 - 6.107)
**Homocysteine**‡						
<13.3 μmol/L	1.000 (reference)	0.893 (0.652 - 1.221)	1.000 (reference)	1.352 (0.993 - 1.841)	1.000 (reference)	1.151 (0.778 - 1.702)
≥13.3 μmol/L	1.272 (0.593 - 2.729)	0.833 (0.460 - 1.508)	0.936 (0.432 - 2.026)	1.518 (0.877 - 2.627)	1.080 (0.680 - 1.716)	1.282 (0.436 - 3.769)

AOR: adjusted by age, gender, hypertension, diabetes mellitus, hyperlipidemia, and smoking status.

† Vitamin B12 434pg/mL and Folate 3.79nmol/L were lower 15% cut-off each level in CAD patients and controls.

‡ Homocysteine 6.42μmol/l were upper 15% cut-off each level in CAD patients and controls.

Supplementary Table 2. Information of PAI-1 polymorphism for PCR-RFLP analysis.

Gene	Polymorphism		Primer sequence	Annealing condition	Cutting enzyme, condition
PAI-1	-844 G>A	Forward	5' - CAG GCT CCC ACT GAT TCT AC - 3'	54°C, 30sec	Xho I, 37C
		Reverse	5' - GAG GGC TCT CTT GTG TCA AC - 3'		
	-675 4G>5G	Forward	5' - CAC AGA GAG AGT CTG GC*C ACG - 3'	53°C, 30sec	Bsl I, 55C
		Reverse	5' - CCA ACA GAG GAC TCT TGG TC - 3'		
	+43 G>A	Forward	5' - TGT CTT CCA GAA CGA TTC CTT CAC - 3'	60°C, 30sec	PshA Ⅰ, 37C
		Reverse	5' - GTT GTC AGC TGG AGC ATG - 3'		

Note: PCR, polymerase chain reaction; RFLP, restriction fragment length polymorphism.

* Altered nucleotide from reference sequence for enzyme recognition.
